# ASO Author Reflections: Optimizing the Oncological Outcome for Locally Advanced Intrahepatic Cholangiocarcinoma

**DOI:** 10.1245/s10434-020-08274-3

**Published:** 2020-03-02

**Authors:** Jun Li, M. Moustafa, J. Meiners, O. Stüben, J. Izbicki, A. Heumann

**Affiliations:** Department of General, Visceral and Thorax Surgery, University Medical Center Hamburg-Eppendorf, Martinistrasse 52, 20246 Hamburg, Germany

## Past

Intrahepatic cholangiocarcinoma (ICC) is an uncommon disease. Nevertheless, a recent review of the SEER database demonstrated that there has been a 10-fold increase in cholangiocarcinoma-related mortality since 1973.^1^ According to recent guidelines, liver resection is the only curative treatment for ICC. An adequate remnant liver volume is one of the limiting factors in ensuring an R0 resection.^2^,^3^ Associating liver partition and portal vein ligation for staged hepatectomy (ALPPS) could increase the resectability in advanced primary and secondary liver malignancies. However, concerns were raised due to the resulting high postoperative morbidity and mortality as well as unknown oncological benefit in non-colorectal metastasis settings.^4^,^5^

## Present

In the registry cohort study from 31 international institutes, 102 patients with locally advanced ICC undergoing ALPPS were recruited.^6^ The R0 resection rate was 85%. The initial high morbidity and mortality rates decreased steadily to a 29% severe complication rate and 7% 90-day mortality in the last 2 years. Multivariate analysis revealed insufficient future liver remnant at stage-2 operation (FLR2) to be the only risk factor for severe complications. A superior overall survival was found in the ALPPS group compared to the propensity score matched palliative chemotherapy group from the SEER database (26.4 months vs 14 months in median). A survival benefit was not confirmed in the subgroup of patients with multifocal ICC. The authors therefore recommend ALPPS to be performed only in ICC patients with a single lesion at this stage and a sufficient FLR2 must be ensured. A high recurrence rate remains an unsolved problem for this disease.

## Future

Generally, locoregional recurrence occurs in 60% and distant recurrence in 30% following liver resection for ICC.^7^ During the period of liver regeneration after hepatectomy, it is anticipated that the remaining circulating tumor cells also receive growth stimulation, which would potentially result in local or distant recurrence.^8^ To achieve a long-term disease-free survival after R0 resection, it is important to control these occult tumor cells before they are stimulated.

This rational provoked our research group to design a neoadjuvant chemotherapy regimen (NEOMAIC trial) in this group of patients, who are going to experience an overwhelming regenerating process under ALPPS or conventional portal vein embolization. Due to the rarity of the disease, the current treatment recommendations in both the adjuvant and neoadjuvant settings are based on a paucity of Phase III trial data. Complicating this further, most of these datasets include patients with gallbladder disease and extrahepatic cholangiocarcinoma.^9^,^10^ A clinical trial limited only to ICC, supported by highly specialized international institutes, would be the best approach to address this issue.
